# Levofloxacin-Induced Hepatotoxicity in Patients With Legionnaires’ Disease: Implications and Management

**DOI:** 10.7759/cureus.51248

**Published:** 2023-12-28

**Authors:** Mohamed Ismail, Nishi Parikh, Merry Zhai, Menna-Allah Elaskandrany, Weizheng Wang

**Affiliations:** 1 Medicine, Rutgers University New Jersey Medical School, Newark, USA; 2 Internal Medicine, Rutgers University New Jersey Medical School, Newark, USA; 3 Internal Medicine, Lenox Hill Hospital, New York, USA; 4 Gastroenterology and Hepatology, Rutgers University New Jersey Medical School, Newark, USA

**Keywords:** hepatotoxicity, infectious diarrhea, shortness of breath (sob), levofloxacin toxicity, legionnaires disease

## Abstract

Legionnaires' disease (LD), caused by the *Legionella* bacterium, primarily manifests as pneumonia and could result in a spectrum of clinical severity. As treatment necessitates the use of antibiotics, levofloxacin, a fluoroquinolone, is a commonly preferred option due to its broad-spectrum activity. However, the potential side effects of levofloxacin, including rare instances of hepatotoxicity, introduce a therapeutic challenge. This case report explores the association between levofloxacin and hepatotoxicity and its implications for treating LD.

## Introduction

Legionnaires' disease (LD), named after its first recognized outbreak at a 1976 American Legion convention in Philadelphia, is a severe form of pneumonia predominantly caused by the bacterium *Legionella pneumophila* [1]. It is one of the critical diseases under the umbrella of atypical pneumonia, which is not typically detected through common laboratory methods or protected against standard pneumonia vaccines [2]. Legionellosis typically presents in two main forms: LD, a severe multisystem illness that includes pneumonia and often requires hospitalization, and Pontiac fever, a self-limiting illness with flu-like symptoms. Many people who develop antibodies to *Legionella* may not exhibit any symptoms. LD has a mortality rate of 8-12%, with increased rates in hospital-acquired cases and elderly individuals [3,4].

Levofloxacin, a third-generation synthetic fluoroquinolone, stands out as a primary therapeutic agent for LD. Its superior lung tissue penetration makes it a top-tier choice for respiratory infections. Compared to other antibiotics, levofloxacin has demonstrated higher cure rates and lower treatment failure rates in LD, further solidifying its position as a primary therapeutic agent [5].

However, amidst its efficacy lie rare yet potential adverse events, notably hepatotoxicity. This raises concern, given the liver abnormalities that may be present in LD [6]. Hepatotoxicity emerges as one of levofloxacin's more severe side effects, ranging from mild transaminase elevations to fulminant hepatic failure. This complication typically appears within the first few weeks of therapy and may present with jaundice, abdominal discomfort, and pronounced fatigue [7]. Fluoroquinolone-associated liver damage has been documented in various case studies and highlighted in numerous reviews. Yet, there is a lack of thoroughly detailed and forward-tracking cases, specifically on liver injury resulting from this antibiotic class [8,9]. We report a case of a 65-year-old male diagnosed with LD, for whom levofloxacin was promptly prescribed. Within three days after starting the treatment, the patient exhibited significant signs of hepatotoxicity. This was evidenced by a substantial elevation in liver enzymes, far exceeding the baseline levels. Following a switch in antibiotic treatment from levofloxacin to azithromycin, there was a notable gradual improvement in the patient's liver enzyme values.

## Case presentation

**Figure 1:**
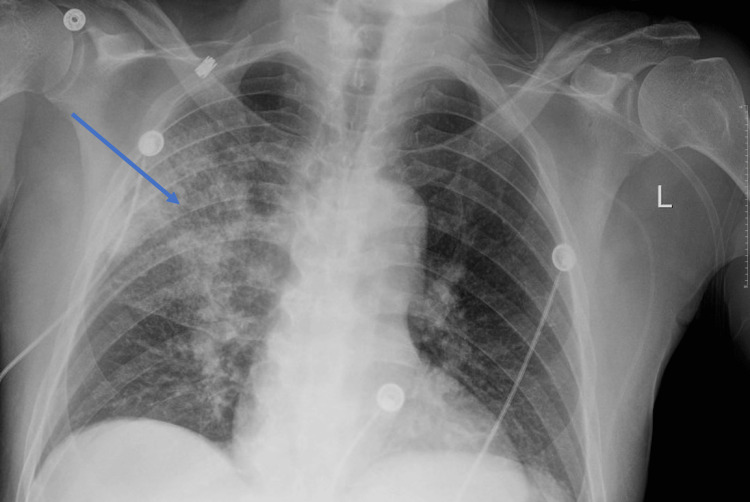
Chest X-ray shows the presence of a right upper lobe consolidation, consistent with pneumonia

A 65-year-old male with a past medical history of hypertension, chronic obstructive pulmonary disease (COPD), tobacco, opioid, and cocaine use disorders presented with fever, weakness, and diarrhea for three days. In the emergency department, the patient was febrile to 104F, hypertensive, tachycardic, saturating 94% on 6 L/min through a nasal cannula, and demonstrated increased work of breathing. His chest X-ray showed right upper lobe interstitial opacities concerning atypical pneumonia (Figure 1). Lab work was significant for a negative respiratory panel, including COVID-19, normal white blood cell count, and procalcitonin elevated to 3.69 ng/mL (reference range ≤0.50 ng/mL). The patient's need for oxygen increased, necessitating an upgrade to bilevel-positive airway pressure (BiPAP) therapy. While receiving oxygen at 10 L/min through a nasal cannula, his saturation levels were observed to be in the range of 83-85%. Consequently, to effectively manage these escalating oxygen demands, his treatment protocol was advanced to include BiPAP support.

The patient received a single dose of cefepime and vancomycin as empirical therapy for suspected sepsis. The initial liver function tests (LFTs) revealed a mild elevation in aspartate aminotransferase (AST) at 45 U/L, while the alanine aminotransferase (ALT) level was within the normal limit at 33 U/L. Notably, the patient did not have a history of alcohol use disorder. Monthly urine drug screens from his addiction medicine appointments showed infrequent alcohol positivity. His baseline LFTs, conducted 10 months before the current hospital admission, indicated normal levels for both AST and ALT, with each enzyme measured at 32 U/L.

On day 2, the patient was found to be positive for *Legionella *urine antigen, and antibiotics were switched to levofloxacin. The patient was weaned off noninvasive ventilation but required a nasal cannula. On day 4, LFTs showed a marked increase: AST 530 U/L and ALT 426 U/L (Table 1). It was suspected that this rapid elevation in liver enzymes was due to levofloxacin hepatotoxicity. As a result, levofloxacin was switched to azithromycin on day 4.

**Table 1:** Liver enzyme trends before and after transitioning from levofloxacin to azithromycin treatment AST: aspartate aminotransferase, ALT: alanine aminotransferase, PO: per oral

Hospital day	Antibiotic	AST (reference: 0-40 U/L)	ALT (reference: 0-41 U/L)	Comments
1	Cefepime 1 g, vancomycin 1 g once	45	33	-
2 (admission)	Levofloxacin 750 mg PO daily	75	38	-
3	Levofloxacin 750 mg PO daily	-	-	No labs were collected
4	Azithromycin 500 mg PO daily	530	426	On this day, labs resulted before the patient was switched to azithromycin
5	Azithromycin 500 mg PO daily	341	431	-
6	Azithromycin 500 mg PO daily	177	342	-
7 (discharge)	Azithromycin 500 mg PO daily	92	250	-

Clinically, the patient did not have jaundice or abdominal tenderness. After switching antibiotics, transaminases began to downtrend, eventually decreasing to AST 92 U/L and ALT 250 U/L (Table 1). The patient was sent home with a prescription for azithromycin to finish a total 10-day course of antibiotics. He was advised to schedule follow-up outpatient appointments with his primary care physician and pulmonologist.

## Discussion

Clinically, LD ranges from a mild febrile illness, known as Pontiac fever, to a severe multisystem disease. Its clinical symptoms overlap with many forms of pneumonia, making early diagnosis somewhat challenging [5]. Identifying atypical extrapulmonary manifestations is essential for timely treatment. Notably, compared to other pneumonias, LD more frequently results in liver dysfunction, hematuria, and proteinuria. The prognosis for LD is favorable if treated promptly, which often requires a more extended treatment duration with specific antibiotics than other pneumonias do. However, the disease can be fatal if left untreated, particularly in those with compromised immune systems or underlying health conditions [10].

Due to its high pulmonary concentration and broad-spectrum activity, levofloxacin is a commonly preferred fluoroquinolone for the treatment of atypical pneumonia caused by the *Legionella* bacterium [8,11]. Generally, levofloxacin and other fluoroquinolones are well tolerated, with common side effects such as gastrointestinal issues, headaches, skin rashes, and allergic reactions. However, they may occasionally lead to more severe side effects, although these occurrences are infrequent. These include QT prolongation, seizures, hallucinations, tendon ruptures, severe hypersensitivity reactions, Stevens-Johnson syndrome, angioedema, photosensitivity, and drug-induced liver injury [8,12].

Beyond the implications of levofloxacin, it's essential to note that LD can independently predispose patients to hepatic aberrations. A sizeable proportion of patients exhibit liver enzyme elevations through direct bacterial invasion, toxin-mediated damage, or immune responses [3]. Furthermore, short-term use of levofloxacin has been shown in studies to increase LFTs by 2-5%. Onset is acute, can start with a hepatocellular pattern of injury, and patients are typically asymptomatic. Although levofloxacin-associated liver injury is uncommon, fluoroquinolones as a drug class are a known cause of liver injury (likelihood score of A) [12]. The suspected mechanism of injury is a hypersensitivity reaction, although patients do not always present with other allergy symptoms such as rash, fever, or eosinophilia. If symptoms are severe or persistent, corticosteroids can be considered; however, most cases are self-limiting [12].

While LD itself may cause liver abnormalities, the severity and rapid onset of our patient's transaminitis suggest a drug-induced etiology. In this scenario, the concomitant use of a potentially hepatotoxic drug complicates clinical management, as the rise in LFTs may be due to LD. However, the patient’s transaminases were not elevated during his initial presentation to the emergency department and only began to increase once levofloxacin was administered. Similarly, the decrease in LFTs after our patient was switched from levofloxacin to azithromycin, as evident on day 5, may indicate that the transaminitis was primarily a result of levofloxacin use and less likely due to LD. This temporal relationship between the duration of levofloxacin use in relation to the onset and resolution of the patient’s transaminitis makes levofloxacin a likely cause. This is supported by an analysis of 12 cases involving hepatotoxicity linked to fluoroquinolones, including ciprofloxacin, moxifloxacin, and levofloxacin, which revealed consistent characteristics. These cases demonstrated a brief latency period ranging from two to nine days and a sudden onset of liver injury, with patients typically showing rapid recovery upon discontinuation of the medication [13]. In addition, the patient lacked comorbidities such as alcohol use disorder and indolent hepatitis that may have caused transaminitis.

Case reports on levofloxacin-associated hepatotoxicity primarily describe male patients above 60 years of age without a pre-existing liver injury. LFTs increased more than 10 times the upper limit of normal in most cases and frequently occurred in association with elevated total bilirubin and alkaline phosphatase. Recovery time averaged from one to three weeks after drug discontinuation [14]. Although we did not observe a concurrent rise in total bilirubin and alkaline phosphatase, our patient’s baseline characteristics fit the patients described in these case studies. Previous case studies raised other factors affecting liver injury, including impaired renal function and a lack of renally dosing medications; however, our patient’s glomerular filtration rate was >60 mL/min [14]. Medications may cause hepatotoxicity, although this was a less likely cause for our patient. He received budesonide, formoterol, amlodipine, losartan, and famotidine, which are not known to cause liver injury or interact with levofloxacin to increase hepatotoxicity.

Although levofloxacin is a commonly prescribed antibiotic for LD, it is important to be aware of its uncommon side effects. Patients with existing liver injuries should be particularly monitored for worsened hepatotoxicity. Even after discontinuing levofloxacin, providers should be wary of delayed presentations of symptoms. In the prospective study of 679 registrants in the drug-induced liver injury network, the median time to symptom onset was four days but ranged from one to 39 days [8]. Although our patient did not have hepatic risk factors predisposing him to drug-induced liver injury, he had poorly controlled COPD and was a current smoker with a 17-pack-year history. It is possible that these respiratory comorbidities increased his risk of more severe manifestations of LD, including subclinical liver injury, which could have made him more susceptible to drug-induced liver injury.

This case underscores the need for clinicians to remain cautious about potential hepatic adverse events when initiating levofloxacin therapy, especially in patients with LD and those with pre-existing liver damage. Routine monitoring of liver enzymes and prompt action upon abnormal findings can significantly reduce the morbidity associated with such adverse outcomes. Thus, as beneficial as levofloxacin is in LD management, it's imperative to weigh the therapeutic advantages against potential hepatic risks.

## Conclusions

Fluoroquinolones rank among the most frequently prescribed antibiotics due to their high oral absorption, straightforward dosing, and extensive antimicrobial range. National guidelines have even suggested them as a first-choice antibiotic treatment. For patients with LD, initiating levofloxacin requires a nuanced understanding of its hepatotoxic potential. Baseline LFTs, followed by regular monitoring during therapy, are essential. For those with significant elevations in liver enzymes or clinical symptoms suggestive of hepatotoxicity, prompt cessation and evaluation are paramount. In these situations, considering alternative therapeutic agents might be necessary.

## References

[1] Fraser DW, Tsai TR, Orenstein W (1977). Legionnaires' disease: description of an epidemic of pneumonia. N Engl J Med.

[2] Marston BJ, Plouffe JF, File TM Jr (1997). Incidence of community-acquired pneumonia requiring hospitalization. Results of a population-based active surveillance Study in Ohio. The Community-Based Pneumonia Incidence Study Group. Arch Intern Med.

[3] Kumar D, Vanani NB, Dobbs J, Jha P (2023). Legionella-induced hepatitis: a case report. Cureus.

[4] Fields BS, Benson RF, Besser RE (2002). Legionella and Legionnaires' disease: 25 years of investigation. Clin Microbiol Rev.

[5] Yu VL, Greenberg RN, Zadeikis N, Stout JE, Khashab MM, Olson WH, Tennenberg AM (2004). Levofloxacin efficacy in the treatment of community-acquired legionellosis. Chest.

[6] Diederen BM (2008). Legionella spp. and Legionnaires' disease. J Infect.

[7] Mithani R, Lam G, Pillai A (2011). Type 2 autoimmune hepatitis… look and you shall find it. Am J Gastroenterol.

[8] Orman ES, Conjeevaram HS, Vuppalanchi R, Freston JW, Rochon J, Kleiner DE, Hayashi PH (2011). Clinical and histopathologic features of fluoroquinolone-induced liver injury. Clin Gastroenterol Hepatol.

[9] Karim A, Ahmed S, Rossoff LJ, Siddiqui RK, Steinberg HN (2001). Possible levofloxacin-induced acute hepatocellular injury in a patient with chronic obstructive lung disease. Clin Infect Dis.

[10] Kao AS, Myer S, Wickrama M, Ismail R, Hettiarachchi M (2021). Multidisciplinary management of Legionella disease in immunocompromised patients. Cureus.

[11] Wolfson JS, Hooper DC (1991). Overview of fluoroquinolone safety. Am J Med.

[12] (2012). LiverTox. Clinical and research information on drug-induced liver injury.

[13] Schloss M, Becak D, Tosto ST, Velayati A (2018). A case of levofloxacin-induced hepatotoxicity. Am J Case Rep.

[14] Panahi L, Surani SS, Udeani G, Patel NP, Sellers J (2021). Hepatotoxicity secondary to levofloxacin use. Cureus.

